# NF-YC3: The master regulator of tomato-arbuscular mycorrhizal symbiotic symphony

**DOI:** 10.1093/plphys/kiae435

**Published:** 2024-08-20

**Authors:** Ritu Singh

**Affiliations:** Assistant Features Editor, Plant Physiology, American Society of Plant Biologists; Department of Plant Science, University of California, Davis, CA 95616, USA

Arbuscular mycorrhizal (AM) symbiosis is a mutualistic interaction between fungi from the Glomeromycotina subphylum and the roots of most terrestrial plants, including many crucial crops. In this relationship, plants supply assimilated carbon to the fungi, which in turn provide essential minerals to the plants (Choi et al. 2018). The development of AM symbiosis is a dynamic process comprising several stages: (1) presymbiotic communication, (2) contact and penetration, (3) outer cortex invasion, (4) arbuscule formation, and (5) vesicle and spore formation. Although these stages occur consecutively at individual infection sites, the overall process in a root system is nonsynchronous. Coordinating this complex development requires intricate and well-regulated signaling mechanisms. Transcriptomic analyses have identified hundreds of differentially expressed genes between mycorrhizal and nonmycorrhizal roots in several plant species (Sugimura and Saito 2017; An et al. 2018; Vangelisti et al. 2018). However, the roles of these genes in AM symbiosis remain unclear, highlighting the requirement for further research into the precise molecular mechanisms regulating AM symbiosis.

A recent study published in *Plant Physiology* by Chien et al. (2024) identified the AM-induced NF-YC3 transcription factor (TF) in tomato and investigated its functional interactions. NF-Y TFs play a pivotal role in AM symbiosis (Hogekamp and Küster 2013; Deng et al. 2022). The authors performed an expression analysis of 13 *NF-YC* genes during AM symbiosis and observed that only *NF-YC3* was significantly upregulated, suggesting it is the major *NF-YC* involved in regulating AM symbiosis. Further, a transient localization study in *Nicotiana benthamiana* leaves suggested that NF-YC3 is mainly localized in the nucleus, implying that NF-YC3 may work as a TF component during AM symbiosis.

To identify the *cis*-regulatory elements responsible for *NF-YC3* expression, the authors analyzed the 3000 bp upstream of the *NF-YC3* gene and identified 5 candidate *cis*-element binding sites. Promoter-GUS (β-glucuronidase) assays showed GUS signals mainly in arbusculated cells driven by NF-YC3 promoter. However, the loss of AM expression was observed in the full-length promoter when the GCC boxes at −502 and −186 positions were mutated. This indicates that *NF-YC3* is primarily induced in arbusculated cells and requires the GCC box for its expression.

Previous reports indicate that the GCC box is in the middle of the *AMCYC-RE* element in the *LjRAM1* promoter, which is directly bound by the CCaMK-CYCLOPS-DELLA complex as a transcriptional activator (Pimprikar et al. 2016). To determine if *NF-YC3* is similarly regulated by the CCaMK-CYCLOPS complex, a transactivation assay combining pNF-YC3, CYCLOPS, and autoactive CCaMK^297^ was performed in *N. benthamiana* leaves. Compared with the empty vector (EV) control, significantly increased reporter activity was observed only when both CYCLOPS and CCaMK^297^ were co-transformed with pNF-YC3. This transactivation effect was impaired when both GCC boxes were mutated, indicating that *NF-YC3* expression can be induced by CYCLOPS in the presence of CCaMK^297^ via GCC boxes.

Since NF-YC3 was primarily expressed in arbusculated cells, Chein and coworkers further investigated the role NF-YC3 in arbuscule development using hairy roots transformed with *nf-yc3* RNAi constructs. *nf-yc3* RNAi roots contained a higher number of smaller arbuscules and fewer large arbuscules compared with EV controls. The overall arbuscule area was also reduced in *nf-yc3* RNAi roots, indicating that *NF-YC3* plays a role in arbuscule development. Moreover, *nf-yc3* RNAi roots showed significantly lower levels of intraradical hyphae (hyphae inside plant root), arbuscule, vesicle, spore, and extraradical hyphae compared with EV. This results in a significant reduction in total colonization level, indicating that *NF-YC3* positively regulates AM symbiosis in tomato.

Further, to identify the potential downstream genes of *NF-YC3*, RNA-seq was performed between *nf-yc3* RNAi and EV roots under mycorrhizal conditions. Transcriptome analysis showed that numerous AM-upregulated genes, including enzymes, kinases, transporters, TFs responsible for arbuscule development, and arbuscule initiation marker genes, exhibited reduced expression in *nf-yc3* RNAi roots compared with EV. Among these, *SbtM1* (arbuscule initiation marker gene), *WRI5a* (*WRINKLED protein*), and *BCP1* (*Blue Copper Protein 1*) were identified as important genes in mediating AM symbiosis (Jiang et al. 2018). Overexpression (OE) NF-YC3 hairy roots were generated to further confirm activation of these genes. *SbtM1*, *WRI5a*, and *BCP1* were significantly upregulated in NF-YC3 OE roots compared with EV under mycorrhizal conditions, suggesting they might be potential downstream genes of NF-YC3.

NF-YC proteins form a functional heterotrimeric complex with 1 NF-YB and 1 NF-YA/other TF before regulating gene expression (Rípodas et al. 2019). To identify putative NF-YB subunits that might interact with NF-YC3, a phylogenetic tree encompassing tomato NF-YB proteins with well-known nodulation or AM symbiosis-related NF-YB was constructed (Hogekamp and Küster 2013). Phylogenetic analysis identified NF-YB5a/b/c/d and NF-YB3a/b as potential interacting partners with NF-YC3. Expression analysis during multiple weeks of AM symbiosis showed that *NF-YB3a* and *NF-YB5c* were significantly induced, while *NF-YB3b* was repressed in mycorrhizal roots. No other *NF-YBs* were regulated by AM symbiosis. Further promoter-GUS assay showed that, unlike *NF-YC3*, the GUS signals of *NF-YB3a*, *NF-YB5c*, and *NF-YB3b* were observed in all cell layers under both mock and mycorrhizal conditions. This suggests NF-YBs might have a more general role than NF-YC3 and NF-YC3 is more likely to be a key component for arbuscule development.

To further verify the interaction of NF-YC3 and 3 NF-YBs, the authors further performed pairwise yeast 2-hybrid and bimolecular fluorescence complementation analysis. The results demonstrated that NF-YB3a, NF-YB5c, and NF-YB3b strongly interact with NF-YC3. Similarly, authors also found that NF-YA3a interacted with NF-YC3; however, the role of NF-YA3a in AM symbiosis remains unclear.

Finally, to characterize the relevance of all 3 NF-YBs in AM symbiosis, single (*nf-yb3a*, *nf-yb5c*, and *nf-yb3b*), double (*nf-yb3a*/*nf-yb5c*), and triple (*nf-yb3a*/*nf-yb5c*/*nf-yb3b*) *NF-YBs* RNAi plants were generated. Only triple (*nf-yb3a/nf-yb5c/nf-yb3b*) RNAi plants showed reduced total fungal colonization levels, implying the functional redundancy among NF-YBs in regulating AM symbiosis.

Overall, this study demonstrates that NF-YC3 is central to the NF-Y complex in tomato-AM symbiosis and positively regulates AM symbiosis (Fig.). The GCC-box is essential for the arbuscule-related expression pattern of *NF-YC3*, which might be regulated by the CCaMK-CYCLOPS complex. The expression of *NF-YB3a*, *5c*, and *3b* is regulated by AM symbiosis; however, the responsible TF remains unclear. After translation, NF-YC3 and NF-YBs interact in the nucleus, potentially forming a trimer complex with NF-YA3a to regulate genes essential for AM symbiosis. The role of NF-YA3a in AM symbiosis needs to be further explored. Knockdown of *NF-YC3* significantly affected the level of intraradical hyphae and arbuscule area. Compared with the loss of 1 NF-YB, the loss of 3 NF-YBs collectively significantly affected the total colonization level, suggesting that functional redundancy of NF-YBs exists.

**Figure.**
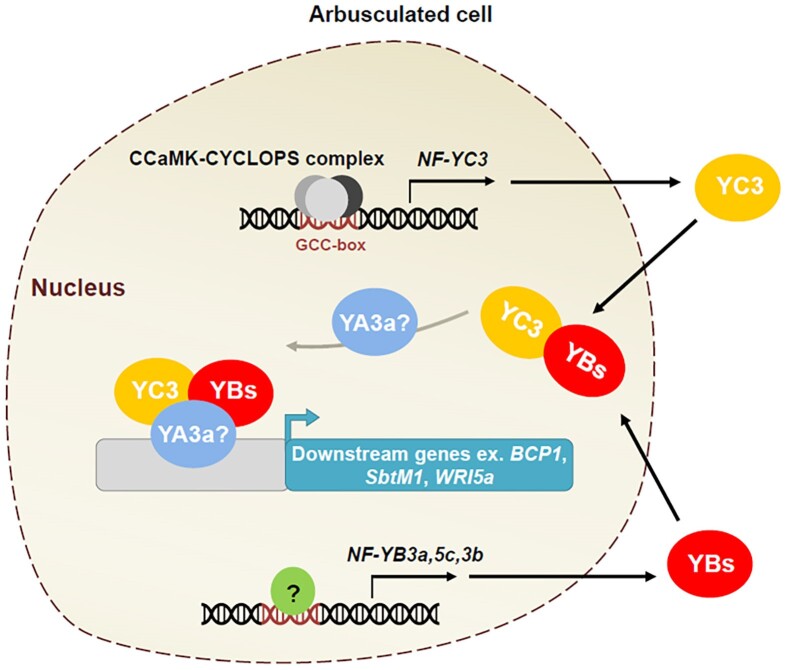
Schematic diagram of NF-YC3 molecular pathways in tomato-AM symbiosis (adapted from Chien et al. 2024, Fig. 8). The CCaMK-CYCLOPS complex binds to the GCC-box and activates *NF-YC3* TF. The *NF-YB3a*, *5c*, and *3b* expression is regulated by AM symbiosis; however, the responsible TF is still unclear. Post translation, NF-YC3 and 3 NF-YBs can interact with each other in the nucleus. Then, NF-YC3 and NF-YB dimer might interact with NF-YA3a to form a trimer complex, which bind to the promoter of downstream genes essential for AM symbiosis. Although NF-YC3 could interact with NF-YA3a in yeast, the role of NF-YA3a in AM symbiosis remains elusive.
